# Bell Shape Curves of Hemolysis Induced by Silver Nanoparticles: Review and Experimental Assay

**DOI:** 10.3390/nano12071066

**Published:** 2022-03-24

**Authors:** Roberto Luna-Vázquez-Gómez, María Evarista Arellano-García, Yanis Toledano-Magaña, Juan Carlos García-Ramos, Patricia Radilla-Chávez, David Sergio Salas-Vargas, Francisco Casillas-Figueroa, Balam Ruiz-Ruiz, Alexey Pestryakov, Nina Bogdanchikova

**Affiliations:** 1 Escuela de Ciencias de la Salud, Campus Ensenada, Universidad Autónoma de Baja California (UABC), Mexicali 21100, Mexico; rluna@uabc.edu.mx (R.L.-V.-G.); yanis.toledano@uabc.edu.mx (Y.T.-M.); juan.carlos.garcia.ramos@uabc.edu.mx (J.C.G.-R.); salasd@uabc.edu.mx (D.S.S.-V.); casillas.francisco@uabc.edu.mx (F.C.-F.); bruiz@uabc.edu.mx (B.R.-R.); 2 Facultad de Ciencias, Campus Ensenada, Universidad Autónoma de Baja California (UABC), Mexicali 21100, Mexico; 3 Research School of Chemistry and Applied Biomedical Sciences, Tomsk Polytechnic University, 634050 Tomsk, Russia; 4 Nanoscience and Nanotechnology Center (CNyN), Campus Ensenada, National Autonomous University of Mexico (UNAM), Mexico City 04510, Mexico; nina@cnyn.unam.mx

**Keywords:** hemolysis, bell-shaped profile, silver nanoparticles, diabetic and healthy donor erythrocytes

## Abstract

The hemolytic activity assay is a versatile tool for fast primary toxicity studies. This work presents a systematic study of the hemolytic properties of Argovit^TM^ silver nanoparticles (AgNPs) extensively studied for biomedical applications. The results revealed an unusual and unexpected bell-shaped hemolysis curve for human healthy and diabetic donor erythrocytes. With the decrease of pH from 7.4 and 6.8 to 5.6, the hemolysis profiles for AgNPs and AgNO_3_ changed dramatically. For AgNPs, the bell shape changed to a step shape with a subsequent sharp increase, and for AgNO_3_ it changed to a gradual increase. Explanations of these changes based on the aggregation of AgNPs due to the increase of proton concentration were suggested. Hemolysis of diabetic donor erythrocytes was slightly higher than that of healthy donor erythrocytes. The meta-analysis revealed that for only one AgNPs formulation (out of 48), a bell-shaped hemolysis profile was reported, but not discussed. This scarcity of data was explained by the dominant goal of studies consisting in achieving clinically significant hemolysis of 5–10%. Considering that hemolysis profiles may be bell-shaped, it is recommended to avoid extrapolations and to perform measurements in a wide concentration interval in hemolysis assays.

## 1. Introduction

Nanotechnology offers numerous metallic nanoparticles (Ag, Zn, Au, Cu, etc.) for their applications in different areas, such as agriculture [1,2,3], veterinary [4,5], textiles [6,7], and medicine [8,9,10,11,12]. Within medicine, nanotechnology is presented with a capacity for use in such important areas as cancer diagnosis and therapy, antibacterial, antiviral, and antifungal agents, bioimaging and molecular diagnosis, drug and gene delivery carriers, etc. [9,10,11,12].

Silver nanoparticles (AgNPs) have been studied more than other nanoparticles for their application in medicine thanks to their broad spectrum of antimicrobial, anticancer, antiviral, and anti-inflammatory properties [13,14,15,16,17].

In our previous publications, we reported the advances in the application of Argovit^TM^ formulation of silver nanoparticles in the treatment of diabetic ulcers [18,19,20], in vitro and in vivo study of cancer [21,22], cyto-y genotoxicity of human lymphocytes [23], ambient toxicity [24], interaction with dendritic cells [25], etc. These studies cover a wide interval of Argovit^TM^ AgNPs concentrations from 0.01 to 30,000 µg/mL. Additionally, our interest in Argovit^TM^ has been attracted by the fact that it has low hemolytic toxicity in relation to human erythrocytes. In our previous work, it was revealed that Argovit™ possesses the lowest hemolytic capacity among 22 other AgNPs formulations [26]. However, in this work, hemolysis induced by Argovit™ AgNPs was studied in a relatively narrow concentration interval (200–800 µg/mL). The absence of complete toxicity studies limits the application of AgNPs, despite their promising prophylactic and therapeutic results obtained on in vitro and in vivo models. Therefore, there was a clear need to perform a systematic study of hemolytic properties of Argovit^TM^ AgNPs in a wider concentration interval (2 to 50,000 µg/mL) than what was studied earlier (200–800 µg/mL).

Therefore, the aim of the present work was to perform a systematic study of the hemolytic properties of Argovit^TM^ AgNPs in a wide interval of concentrations. For this, a variation of parameters important for biomedical applications, such as pH, erythrocytes source (healthy and diabetic donors), and compound type (AgNPs and AgNO_3_), were performed. The results obtained in this work revealed an unusual and unexpected bell shape of the hemolysis curve. A meta-analysis permitted to reveal the reason why previously this bell-shaped curve of hemolysis induced by AgNPs was registered (but not discussed) only in one publication. The importance of the bell shape for hemolysis data extrapolations is discussed. 

## 2. Materials and Methods

### 2.1. Characterization of AgNPs Formulation Argovit™

Argovit™ AgNPs were provided by Dr. Vasily Burmistrov (Vector-Vita Scientific and Production Center, Novosibirsk, Russia). Argovit™ is a stable water suspension of 200 mg/mL of AgNPs (20% *w*/*w*). The suspension containing 1.2% *w/w* of metallic silver was stabilized with 18.8% *w*/*w* polyvinylpyrrolidone (PVP 12.6 ± 2.7 kDa), and the remaining 80% of the formulation was distilled water. AgNPs have a spherical shape with a size distribution from 1 to 90 nm in diameter, with an average of 35 ± 12 nm, a hydrodynamic diameter of 70 nm, a ζ potential of −15 mV, and a plasmonic resonance at 420 nm [27]. Argovit™ AgNPs have been widely used in medical [20,28,29], veterinary [30], and industrial applications, having the corresponding certificates of use [31,32].

### 2.2. Solutions

Argovit^TM^ AgNPs and AgNO_3_ were prepared at 0.001, 0.011, 0.028, 0.056, 0.111, 1.113, 5.565, 11.129, 22.258, and 27.823 mM of Ag content, at which the erythrocytes were exposed. This corresponds to 2 to 50,000 µg/mL of AgNPs.

AgNO_3_ (St. Louis, MO, USA 2091-39-25G) was selected as a source of Ag^+^ ions due to its high water solubility. Triton X-100 solution was prepared as a positive control, mixing 20 mL of Triton X-100 in 80 mL of distilled water. PBS solutions adjusted to pH = 7.4, 6.8, 6.2, and 5.6 were used as negative controls. 

### 2.3. Erythrocyte Suspensions

For erythrocyte suspensions, 25 mL of venous blood was drawn by vacuum phlebotomy followed by centrifugation at 500× *g* for 5 min. Plasma was discarded, and red blood cells were washed three times with sterile NaCl 150 mM physiological solution. A final wash was carried out with PBS buffer solution at pH = 7.4 under the same conditions. The erythrocyte pack obtained (1 mL) was diluted in 49 mL of PBS at pH 7.4, 6.8, 6.2, and 5.6, achieving a 1:50 erythrocyte stock suspension for each pH level [33].

### 2.4. Hemolysis Test

Triplicates of each test were assembled in 1.5 mL conical bottom tubes, with AgNPs Argovit™ and AgNO_3_ at the concentrations previously described. A negative control (PBS) and a positive control (Triton X-100) were also used. The assay was set up to achieve a final volume of 1000 µL in each trial by adding 50 µL of the test agent to 950 µL of the 1:50 red cell stock suspension. All tubes were incubated simultaneously at 37 °C for 2 h, shaking them by gentle inversion once every half hour. Then, the tubes were centrifuged at 500× *g* for 5 min, and 100 µL of the supernatant was transferred to 96-well plates to obtain absorbance readings at 450 nm in a 96-well ControLab EliRead spectrophotometer (RT-21007) (EliRead, KontroLab, Italy). The hemolysis percentages were calculated using the formula:

$$\%H=100*\left[\frac{\mathrm{Ap}-\mathrm{Ac}\left(-\right)}{\mathrm{Ac}\left(+\right)-\mathrm{Ac}\left(-\right)}\right]$$

where: Ap = absorbance of the sample; Ac(+) = absorbance of the positive control; Ac(−) = absorbance of the negative control with erythrocytes.

### 2.5. Ethical Considerations

This study complies with Mexican Research Regulations and the Declaration of Helsinki. Each participant provided their informed consent before sampling. 

### 2.6. Donors

Healthy donor erythrocytes (HDE) were obtained from a normocytic individual with fasting glucose of 90 mg/dL. Diabetic donor erythrocytes (DDE) were obtained from a normocytic individual with fasting glucose of 140 mg/dL and glycosylated hemoglobin (HbA1c) of 10%.

### 2.7. Statistical Analysis

A generalized regression analysis (General Regression Model (GRM)) was performed, due to the heterogeneity of the variances, using the software StatSoft ™ Statistica V.13.3 1984–2017 (TIBCO Software Inc., Palo Alto, CA, USA) to estimate the hypotheses on the effects included in the design: pH in the medium (7.4, 6.8, 6.2, and 5.6), two sources of silver (AgNO_3_ and AgNPs), donor condition (healthy and diabetic), and the ten concentrations, which allowed estimating and testing hypotheses about the effects included in the final model [34]. The main results and some covariates were plotted with GraphPad Prism 9.2.0. (332) (San Diego, CA, USA).

$$\%\mathrm{Hemolysis}=\beta_{0}+\beta_{1}\left(\mathrm{pH}\right)+\beta_{2}\left(\mathrm{Ag}\;\mathrm{Source}\right)+\beta_{3}\left(\mathrm{Donor}\;\mathrm{condition}\right)+\beta_{4}\left(\mathrm{Conc}.\right)\ldots +..\beta_{\mathrm{covariates}}+\epsilon$$

## 3. Results

### 3.1. Bell-Shaped Hemolysis Profile 

**Figure 1:**
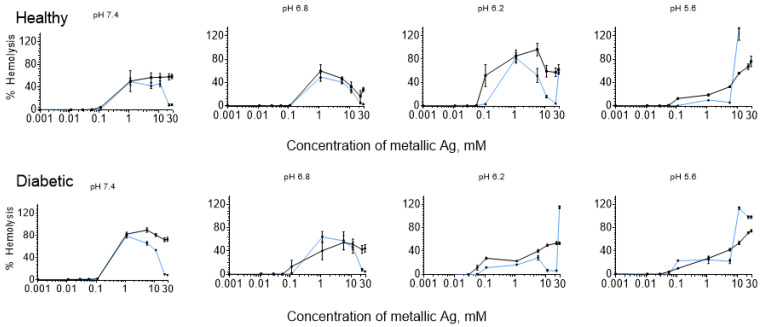
Hemolysis induced by AgNPs and AgNO_3_ at different pH for HDE and DDE: AgNPs correspond to blue lines and AgNO_3_ to black lines.

**Figure 2:**
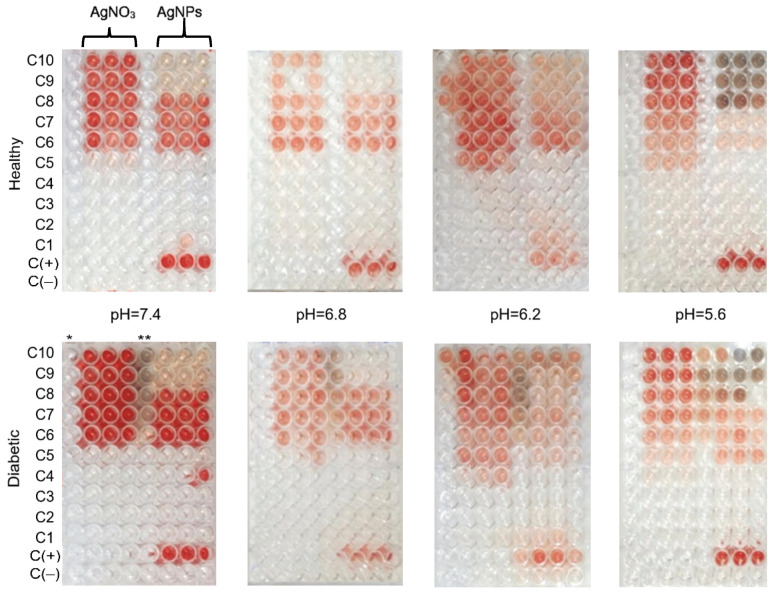
Photograph of 96-well plates of hemolyzed samples with triplicates for diabetic and healthy donor erythrocytes at different pH and concentrations. Concentrations from C1 to C10 for AgNPs and AgNO_3_, at 0.001, 0.011, 0.028, 0.056, 0.111, 1.113, 5.565, 11.129, 22.258, and 27.823 mM, correspond to metallic silver concentrations, respectively. Positive (C+ Triton X-100) and negative (C− with erythrocytes) controls are included. (*) Represents AgNO_3_ without erythrocytes and (**) AgNPs without erythrocytes.

The hemolytic profiles of erythrocytes from healthy and diabetic donors, ex vivo exposed to Argovit™ AgNPs and AgNO_3_ (used as a source of Ag^+^ ions) at different pH, are shown in Figure 1 (corresponding photographs are presented in Figure 2). The vertical axis shows the percentage of hemolysis induced by both substances analyzed, and the horizontal axis represents the concentration of metallic Ag on a logarithmic scale. 

**Figure 3:**
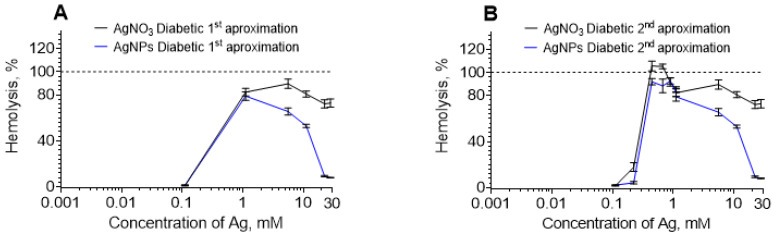
Hemolysis induced at pH 7.4 in DDE: (**A**) first approximation with two experimental points and (**B**) second approximation with six experimental points in the interval 0.1–1 mM. Hemolysis for AgNPs is depicted in blue lines and AgNO_3_ in black lines.

In Figure 1, one unusual fact worthy of discussion was noted. The hemolysis with bell-shaped profiles did not reach 100%. We supposed that this was due to the abrupt change from a 0.1 to 1 mM concentration. Therefore, a more detailed study in this concentration interval was carried out for AgNPs and AgNO_3_ at pH 7.4 for DDE (Figure 3), which revealed that 100% hemolysis was reached between 0.1 and 1 mM for both compounds. Further experiments could determine the more precise maximum position for other curves of Figure 1. 

### 3.2. DDE and HDE

**Figure 4:**
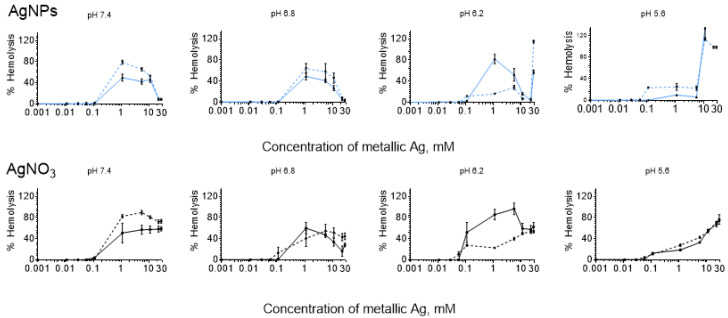
Hemolysis induced by AgNPs at pH 7.4, 6.8, 6.2, and 5.6 in DDE (dotted) and HDE (continuous), blue lines, and AgNO_3_ in DDE (dotted) and HDE (continuous), black lines.

Under the studied conditions in most cases, the hemolysis caused by AgNPs and AgNO_3_ for DDE was slightly higher than for HDE (Figure 4). This result is consistent with the osmotic fragility of DDE caused by hyperglycemia [35,36]. However, the data at pH 6.2 were the exception (Figure 4). This effect is apparent. At transition pH 6.2, in healthy erythrocytes, hemolysis profiles still correspond to more neutral pH (7.4 and 6.8) and have a profound bell shape. In contrast, more sensitive DDE hemolysis profiles already correspond to acidic conditions (pH 5.6) and have a low step profile with a following sharp increase.

The surprising fact of the present work is that a hemolysis curve with a bell shape was observed for 6 out of 8 cases of hemolysis induced by AgNPs (Figure 1), naturally leading to the following question: Was this shape observed in previously published works dedicated to the study of human hemolysis caused by AgNPs? To answer this question, a meta-analysis was performed.

### 3.3. Meta-Analysis

**Figure 5:**
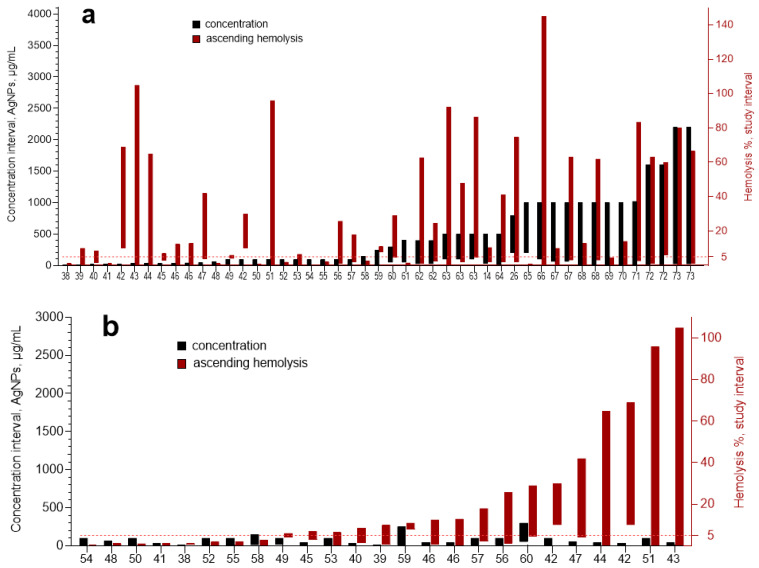

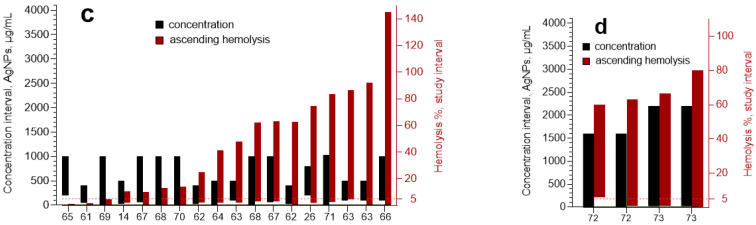
Hemolysis percentage range (red columns) and corresponding interval of AgNPs concentrations (black columns). General view for 47 AgNPs formulations ordered in accordance with the increase in the maximum concentration of AgNPs (**a**). Three segments of Figure 5a ordered according to maximum hemolysis: 25 formulations studied up to 250 µg/mL (**b**), 18 formulations studied up to 1000 µg/mL (**c**), and 4 formulations studied up to 3000 µg/mL (**d**). References of cited publications are indicated on the abscissa. Adapted from Refs. [38,39,40,41,42,43,44,45,46,47,48,49,50,51,52,53,54,55,56,57,58,59,60,61,62,63,64,65,66,67,68,69,70,71,72,73].

**Figure 6:**
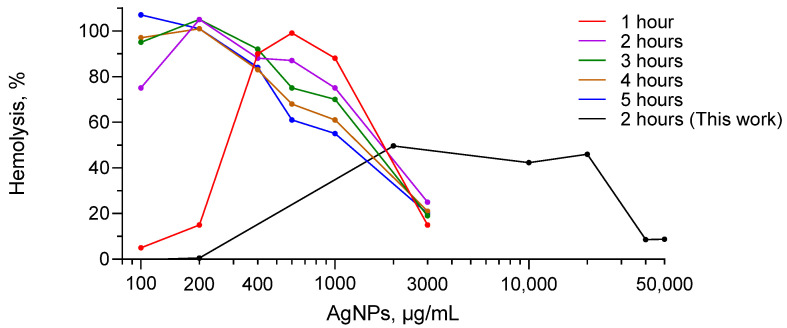
Human erythrocytes hemolysis induced by AgNPs at pH 7.4: data Adapted from Ref. [37] (colored curves) and data of the present work (black curve).

We found previously published data on hemolysis of human erythrocytes induced by 52 different AgNPs formulations. Results of the meta-analysis for hemolysis in human erythrocytes induced by 47 different formulations of AgNPs are graphically represented (Figure 5). Table 1 presents four works dedicated to hemolysis caused by AgNPs, which are not included in Figure 5. The reasons why they were not included are also indicated in Table 1. The hemolytic behavior of AgNPs formulation from [37] is not included in Figure 5 nor in Table 1, instead, it is presented separately in Figure 6 due to the fact that this work is the only work presenting a descending hemolysis aside from our work. It is important to emphasize that in the case of all formulations presented in Figure 5, the hemolysis increased with the AgNPs concentration; in other words, only ascending hemolysis was observed. While for formulations of Figure 6, hemolysis curves are bell-shaped or descending. 

First, we briefly explain the reasons why it was impossible to include the results of the four publications in Table 1. These reasons are: the concentration of AgNPs for which hemolysis was measured is not indicated [74], hemolysis data are presented in mg/dL without indication of initial erythrocyte concentration [75], data of AgNPs are presented in µM (it is impossible to convert it to µg/mL due to the fact that AgNPs are not molecules) [76], and hemolysis data presented in the description and in corresponding figures are inconsistent (with a ten-fold difference) [77].

Figure 5a presents a general view for 47 AgNPs formulations ordered in accordance with the increase in the maximum AgNPs concentration. It is expected that when the maximum studied AgNPs concentration increases, the hemolysis also increases. However, this was not the case (Figure 5a). The hemolysis did not correlate with the maximum AgNPs concentration. It could be a consequence of the big difference in the hemolytic capacity of different AgNPs formulations. It is known that parameters such as size, stabilizer nature, electrical charge (ζ potential), hydrodynamic diameter, and the metallic silver molar ratio to the stabilizer, among others, influence the toxicity of AgNPs [23,24,26].

Results of the meta-analysis showed that among the 47 formulations presented in Figure 5, none had the bell-shaped hemolysis curve. In fact, only four formulations of hemolysis reached more than 90%. In most hemolysis assays, the primary goal is to measure the specific concentration at which significant hemolysis (5–10%) begins [78]. For 23 formulations (among 47), hemolysis was measured not higher than 10%, and for 10 of them, even 5% hemolysis was not achieved (Figure 5a). This indicates that the practical interest of studies sets the limitations for the experiments. 

However, we managed to find a bell-shaped curve for one AgNPs formulation (for 1 h exposure to AgNPs) (Figure 6, red curve) [37]. Moreover, for this AgNPs formulation, the hemolysis corresponding to 2, 3, 4, and 5 h of exposure decreased with the AgNPs concentration [37] (Figure 6). For these exposure times, probably only the descending parts of bell-shaped hemolysis curves were observed, and to reveal the complete bell-shape profile, the hemolysis should be studied at lower AgNPs concentrations. These results correlate with our bell-shaped curve for a 2 h exposure time for HDE at pH 7.4 presented in Figure 6 (black curve). 

We did not perform a profound analysis of the published results for hemolysis of erythrocytes in animals. Nevertheless, after seeing quite a number of works carried out for animals, we managed to find a publication reporting that hemolysis induced by rat and rabbit erythrocytes studied in a wide range of concentrations of AgNO_3_ and freshly prepared AgCl, respectively, shows a bell-shaped profile [79]. In this case, hemolysis rose up to 100% then decreased to 40% and 10% (when AgNO_3_ and AgCl concentrations increased) for rats and rabbits, respectively.

We found a bell-shaped hemolysis profile in only two works [37,79]. Why was this bell shape for the hemolysis curve only reported in very few works? Why did researchers not reveal it more often? The most reasonable answer might be the following. As it was mentioned above, in most hemolysis experiments, the main goal is to measure the specific concentration at which substantial hemolysis starts, which corresponds to 5–10% [78]. Therefore, the practical interest of the research sets the limitations for experiments, and thus researchers do not study hemolysis in a wide interval of concentrations. In order to observe a bell-shaped curve, it is necessary to conduct studies in a fairly wide range of concentrations. In our work, AgNPs concentrations varied 25,000-fold, from 2 to 50,000 µg/mL; in [37], concentration varied 30-fold, from 100 to 3000 µg/mL, and in [79], 250-fold, from 0.02 to 5 mM (calculated for metallic silver). Our systematic work in a wide concentration interval (4 orders of magnitude), and variation of parameters important for biomedical applications (pH, hemolytic agent, erythrocyte source), allowed us to register the bell-shaped curves repeatedly, reliably confirming bell shape reproducibility. 

## 4. Discussion

### 4.1. Bell-Shaped Hemolysis Profile 

Surprisingly, the curves passing through a well-defined maximum were registered for hemolysis caused by AgNPs at pH 7.4 and 6.8 in both HDE and DDE (Figure 1, blue lines). The segment of the hemolysis curve where hemolysis increased, for AgNPs and AgNO_3_, was the same, but at concentrations ≥1 mM, hemolysis of AgNO_3_ practically remained constant (except for the case of pH 6.8 in HDE) (Figure 1). Consequently, at high concentrations, AgNO_3_ was slightly more hemolytic than AgNPs. 

For pH 5.6, the profile of the hemolysis curves for AgNPs and AgNO_3_ drastically changed (Figure 1), which certainly indicates a change in the mechanism of hemolysis. For AgNPs, the bell shape changed to a step shape with a subsequent sharp increase, and for AgNO_3_ it changed to a usual gradual increase. From Figure 1, it is apparent that pH 6.2 represents a transitional state. While for HDE at pH 6.2, the hemolysis curves were like the corresponding curves at pH 7.4 and 6.8, for DDE, they were more like the profiles corresponding to pH 5.6. 

### 4.2. The Possible Reason for the Bell-Shaped Hemolysis Profile 

The study of mechanisms of the processes occurring during hemolysis can explain the bell-shaped profiles, but this requires a series of future experiments. Nevertheless, the approaches presented below could help to guide these future experiments. Erythrocytes-based experimental systems, like many other biological models, are complex. In part, it is because of their chemical content. They include erythrocytes and dilution media, which maintains erythrocytes in an environment similar to human blood. In addition, they contain substances under test, in our case, the AgNPs and AgNO_3_. Below are some clues to better comprehend the complexities of the erythrocytes-based hemolysis experimental system:

AgNPs complexity. Hemolysis experiments are carried out in an atmosphere containing oxygen, which can oxidize the metallic silver of AgNPs to form Ag^+^ silver ions. In solutions containing AgNPs, there is always a balance between AgNPs and Ag^+^ ions, which depends on the effectiveness of the protection of metallic silver by stabilizers, AgNPs size, ζ potential, etc.

Erythrocyte complexity. Erythrocytes have membrane proteins with thiol groups that interact with Ag^+^ or AgNPs to produce sulfides or other silver compounds [80]. The interaction of Ag^+^ with erythrocyte ubiquinone can cause uncoupling of sodium and potassium ion transport [81].

Experimental liquid medium (PBS) complexity. The medium applied for the hemolysis study contains chlorides and phosphates: 137 mM NaCl, 2.7 mM KCl, 10 mM Na_2_HPO_4_, and 1.8 mM KH_2_PO_4_, which can also react with Ag^+^ to form very poorly soluble silver chlorides and phosphates.

Taking into account the complexities mentioned above, in our view, one of the most probable explanations for the drop in hemolysis occurring with the increase of Ag concentration may be the aggregation of AgNPs when hemolysis reaches a specific level. Hemolysis usually decreases as the size of AgNPs increases [82,83]. Agglomeration of AgNPs occurs when the ionic strength of the solution increases, mainly when 2^+^ charged ions such as Mg^2 +^ and Ca^2+^ are formed and released from erythrocytes during hemolysis [84]. Other reasons can also be considered: the formation of AgCl colloids due to the interaction of Ag^+^ ions of AgNO_3_ or AgNPs with chlorides of the experimental liquid medium [85], the interaction of Ag^+^ with ubiquinone of erythrocytes [81], etc. Any explanation needs experimental support and presents a challenge for further studies.

### 4.3. Influence of pH

The pH influence on the hemolysis profile does not contradict the proposed explanation of the hemolysis decrease by AgNPs aggregation. In [86], for AgNPs stabilized with (BH4)^+^ with an initial AgNPs diameter of 26 nm, it was found that a pH change in the interval of 7 to 5 led to significant aggregation of AgNPs. The authors provide the following explanation for this experimental phenomenon: In neutral solutions, AgNPs are stable due to their repulsion caused by electrostatic force associated with their negative charge (ζ potential of Argovit^TM^ AgNPs is −15 mV). In acidic solution, positively charged protons are attracted to negatively charged AgNPs, reducing their negative charge or completely neutralizing them. This causes a decrease or disappearance of the repulsion between AgNPs, which leads to their rapid aggregation. Our results are consistent with the results of [86], where it was shown that the stability of AgNPs is reduced due to their aggregation when pH decreases from 8 to 5. Figure 1 shows that at the transition from pH 7.4 and 6.8 to pH 5.6, a drastic change in the shape of the hemolysis profiles was observed. Considering the results of [86], it is possible to suppose that this change occurred due to easier and faster aggregation of AgNPs when lowering pH to 5.6.

The hemolysis profile for HDE or DDE at pH 6.2 is characterized by maximum and a following increase. As suggested above, the first increase can be due to hemolysis caused by original AgNPs, and the subsequent hemolysis decrease is related to the aggregation of these initial AgNPs. It is well-known that the toxicity of AgNPs decreases with their size, including hemolytic capacity [87]. Therefore, aggregated AgNPs with larger sizes are less hemolytic, and hemolysis induced by them occurs at higher Ag concentrations than those generated by smaller original AgNPs (Figure 1). In acid solutions at pH 5.6, due to easier and faster aggregation of AgNPs, probably only a tiny part of AgNPs do not aggregate. Therefore, maximums associated with non-aggregated AgNPs are low intensive, and the hemolysis is mainly due to aggregated AgNPs. 

The obtained results indicated that pH change (pH 7.4—outside the erythrocyte, pH 6.8, 6.2, and 5.6—within early, mature, and late endosomes) significantly influences the hemolysis profile induced by either AgNPs or AgNO_3_. A bell-shaped hemolysis profile is more likely to be observed in neutral solutions than acid ones.

### 4.4. Hemolysis Caused by AgNO_3_

For hemolysis caused by AgNO_3_, usual hemolysis profiles (smooth growth or growth with access to a plateau) were observed in most cases (Figure 1). However, the hemolysis profile at pH 6.2 and 6.8 in HDE represented exceptions, where a profile with a bell shape was observed. In [55], it was shown that Ag^+^ ions of AgNO_3_ in contact with biological systems (bacterial supernatant) are reduced to AgNPs, and the degree of their reduction significantly depends on the pH. Varying pH from 2 to 11, the maximum reduction occurred at pH 8 [55]. Probably in our case, the Ag^+^ ions to AgNPs reduction in contact with HDE also occurred, and the optimal pH corresponds to pH 6.2 and 6.8. It is worth noting that only an Ag^+^ partial reduction took place because AgNO_3_ induced a hemolysis decrease to only 16% and 57% at pH 6.8 and 6.2, respectively, and hemolysis did not fall as low as in the case of AgNPs (down to 4%).

Results of the meta-analysis (Figure 5a) showed that for 11 formulations, hemolysis did not reach 5%, which is considered a hemolytic level [78], 28 formulations of AgNPs achieved less than 40%, and for 17 formulations, hemolysis reached from 40% to less than 100%. Only for two formulations was hemolysis studied up to 100% (Figure 5a), however higher concentrations were not investigated. However, it is possible that, if these experiments had continued at higher concentrations, hemolysis could be maintained at 100% or could go through a maximum (bell shape). As shown in Figure 6, we could find in the literature only a single work that studies the hemolysis caused by AgNPs in human erythrocytes after reaching 100% hemolysis by further substantially increasing the concentration of AgNPs [37].

**Figure 7:**
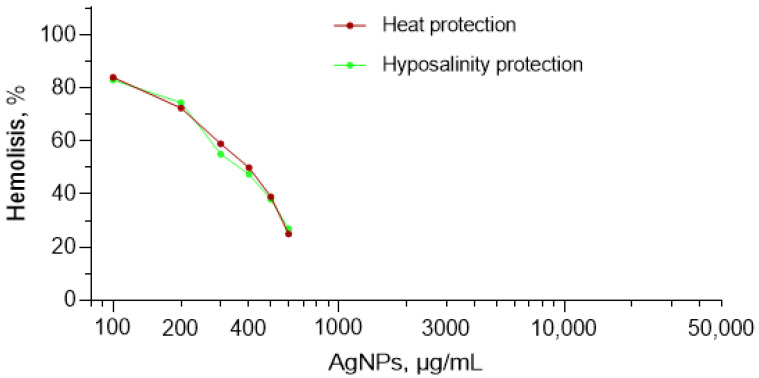
Hemolysis induced by AgNPs under conditions of elevated temperature (56 instead of 37 °C physiological temperature) and hyposalinity (0.1 instead of 1.5 M physiological concentration). The data were Adapted from Ref. [88].

In Figure 7, interesting results on the hemolysis induced by AgNPs under conditions of elevated temperature (56 instead of 37 °C) and hyposalinity (0.1 instead of 1.5 M) are presented. These conditions are already severe enough to induce hemolysis. It would be expected that with the addition of AgNPs, hemolysis will increase. Instead, with AgNPs’ addition, descending hemolysis was observed (Figure 7). The authors named this effect as protection from heat-induced hemolysis and hyposalinity-induced hemolysis, considering that in both cases, AgNPs prevent lysis of the erythrocyte membrane. Results of this work permit suggesting that these protective properties are due to the fact that at high temperature and low salinity, hemolysis induced by AgNPs is presented by a descending branch (decrease with AgNPs concentration).

**Figure 8:**
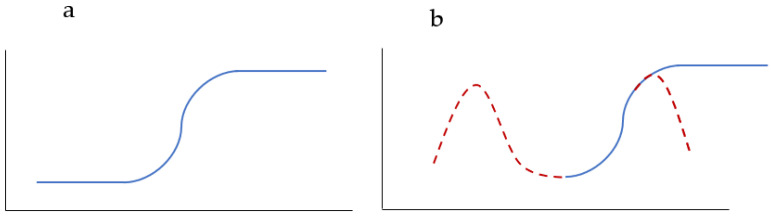
Schematic representation of hemolysis curves for: the current paradigm, where hemolysis begins to increase at some concentrations, then reaches 100% and remains at a 100% plateau with a further concentration increase (**a**), and for cases including the possibility of bell-shaped hemolysis profiles (**b**).

In Figure 8, two types of hemolysis curves are schematically presented. Figure 8a represents the current paradigm, where hemolysis begins to increase at some concentrations, then reaches 100% and remains at a 100% plateau with a further concentration increase. Our results and the results of [34] confirm that in the hemolysis curve, a maximum can be observed. Therefore, it is not possible to say a priori what the AgNPs concentration increase will lead to, either to an increase of hemolysis, or vice versa (Figure 8b). It will depend on the studied concentration, either laying on the ascending branch or descending branch of the bell-shaped hemolysis curve (Figure 8b). In the case of the current paradigm for the hemolysis curve, if one datum for hemolysis is obtained, anyone can extrapolate the results as follows: at lower concentrations, the hemolysis will be lower, at higher concentrations, hemolysis will increase, and after reaching 100%, it will plateau (Figure 8a). Taking into account the possibility of a bell-shaped hemolysis profile (Figure 8b), this extrapolation would not be correct because it can be expected that at lower concentrations, the hemolysis can increase, and at higher concentrations, it can decrease. Hence, extrapolation of results is not recommended and, instead, hemolysis studies should be performed in a wide concentration interval.

Further studies should show whether this bell-shaped profile is observed only for Ag-containing compounds, as was shown for AgNPs, AgNO_3_, and freshly prepared AgCl in [37,79] and in the present work, or whether this shape can be observed for other substances. For what kind of substances is it observed? The possibility of hemolysis with a bell-shaped profile should be considered in the hemolysis test protocols. Future research is also needed to clarify the mechanisms of hemolysis with a bell-shaped profile for Ag-containing compounds.

## 5. Conclusions

This work presented a systematic study of the hemolytic properties of Argovit^TM^ AgNPs, varying four orders of magnitude of AgNPs concentration and varying the parameters important for biomedical applications, such as pH, erythrocytes source (healthy and diabetic donors), and compound type (AgNPs and AgNO_3_). The results obtained in this work revealed an unusual and unexpected bell-shaped hemolysis curve. The bell-shaped profile was observed for human healthy and diabetic donor erythrocytes. With the decrease of pH from 7.4 and 6.8 to 5.6, the hemolysis profiles for both silver compounds changed dramatically. For AgNPs, the bell shape changed to a step shape with a subsequent sharp increase, and for AgNO_3_ it changed to a gradual increase. Explanations of these changes based on the aggregation of AgNPs due to the increase of the proton concentration were suggested. For most of the tested pH values and Ag concentrations, the AgNPs- and AgNO_3_-induced hemolysis of diabetic donor erythrocytes were slightly higher than those of healthy donor erythrocytes, which was explained by the greater fragility of diabetic donor erythrocytes compared to healthy donor erythrocytes. The meta-analysis revealed that a bell-shaped hemolysis profile was reported for only one AgNPs formulation (out of 48), but this was not discussed. We explain this scarcity of data with bell shape profile by the following reason. The dominant goal of all studies (considered in meta-analysis, Figure 5) was to achieve clinically significant hemolysis of 5–10%, and not to study a complete hemolysis profile. Considering that hemolysis profiles may be bell-shaped, it is recommended to avoid extrapolations and to perform measurements in a wide concentration interval in hemolysis assays.

## Figures and Tables

**Table 1:** Publications with data excluded from the meta-analysis.

References	Reason of Exclusion
[74]	The concentration of AgNPs is not indicated.
[75]	Data of hemolysis are presented in mg/dL, and initial erythrocyte concentration is absent.
[76]	Data of AgNPs are presented in µM. It is impossible to convert to µg/mL due to the fact that AgNPs are not molecules.
[77]	Hemolysis data presented in description and in corresponding figures are inconsistent (with a ten-fold difference).

## Data Availability

The data presented in this study are available on request from the corresponding author.
